# Supplementary material for: Patterns of Intron Gain and Loss in Fungi
Source: PLoS Biol. 2004 Nov 30;2(12):e422. doi: 10.1371/journal.pbio.0020422 (PMC532390; doi:10.1371/journal.pbio.0020422)
Supplement: Table S1 — Also available at http://genes.mit.edu/NielsenEtAl/. (4.3 MB ZIP). [file pbio.0020422.st001.zip › NielsenEtAl/html/449.html]

AN1126.1.NCU08340.1.MG04438.1.FG01014.1


```
 CLUSTAL W (1.82) Multiple Sequence Alignments - Introns Inserted


Sequence 1: MG04438.1	183 aa
Sequence 2: FG01014.1	183 aa
Sequence 3: AN1126.1	183 aa
Sequence 4: NCU08340.1	185 aa
Alignment Length: 186 aa
Number Identitical Residues: 155 aa
Alignment Score (without introns) 6185


MG04438.1 	MG--LSIS-KLFDRLWGKKEMRILMVGLDAAGKTTILYKLKLGEIVTTIPTIG1FNVETV
NCU08340.1	MGNTLSIFGKLFDGLFGKKEMRILMVGLDAAGKTTILYKLKLGEVVTTIPTIG1FNVETV
FG01014.1 	MG--LAFS-KLFDRLWGKKEMRILMVGLDAAGKTTILYKLKLGEIVTTIPTIG1FNVETV
AN1126.1  	MG--LAIS-KLFDRLWGKKEMRILMVGLDAAGKTTILYKLKLGEIVTTIPTIG1FNVETV
          	**  *::  **** *:****************************:******** ******

MG04438.1 	EYKNIQFTVWDVGGQDKIRPLWRHYFQNTQGIIFVVDSNDRDRIVEAREELQRMLNEDEL
NCU08340.1	EYKNIQFTVWDVGGQDKIRPLWRHYFQNTQGIIFVVDSNDRDRVVEAREELQRMLNEDEL
FG01014.1 	EYKNIQFTVWDVGGQDKIRPLWRHYFQNTQGIIFVVDSNDRDRIVEAREELQRMLNEDEL
AN1126.1  	EYKNIQFTVWDVGGQDKIRPLWRHYFQNTQGIIFVVDSNDRDRIVEAREELQRMLNEDEL
          	*******************************************:****************

MG04438.1 	RDAILLVFANKQDLP0NAMNAAEITDKLGLHSLRQRAW0YIQSTCATSGDGLYEGLEWLA
NCU08340.1	RDALLLVFANKQDLP0NAMNAAEITDKLGLSSLRQRSW0YIQATCATTGDGLFEGLDWLS
FG01014.1 	RDAILLVFANKQDLP0NAMNAAEITDKLGLHSLRQRAW0YIQSTCATSGDGLYEGLEWLA
AN1126.1  	RDALLLVFANKQDLP0NAMSPAEITQQLGLQSLTRRPW0YIQSTCATTGDGLYEGLEWLA
          	***:*********** ***..****::*** ** :*.* ***:****:****:***:**:

MG04438.1 	TTLRKAGHQ
NCU08340.1	TELKKKSP-
FG01014.1 	NTLRKAGHQ
AN1126.1  	ETLRKTGRD
          	  *:* . .
```
